# Supplementary material for: Crosstalk between CD64+MHCII+ macrophages and CD4+ T cells drives joint pathology during chikungunya
Source: EMBO Mol Med. 2024 Feb 8;16(3):11. doi: 10.1038/s44321-024-00028-y (PMC10940729; doi:10.1038/s44321-024-00028-y)
Supplement: Supplementary file 1 — Appendix [file 44321_2024_28_MOESM1_ESM.pdf]

Appendix

Crosstalk between CD64⁺MHCII⁺ macrophages and CD4⁺ T cells drives joint pathology during chikungunya

Authors: Fok-Moon Lum[1,†,*], Yi-Hao Chan[1,†], Teck-Hui Teo[1], Etienne Becht[2,3], Siti Naqiah Amrun[1], Karen W.W. Teng[2], Siddesh V. Hartimath[4], Nicholas K.W. Yeo[1], Wearn-Xin Yee[2], Nicholas Ang[2], Anthony M. Torres-Ruesta[1], Siew-Wai Fong[1], Julian L. Goggi[4], Evan W. Newell[2,3], Laurent Renia[1,2,5,6], Guillaume Carissimo[1,7] and Lisa F.P. Ng[1,8,9,10,11,*]

*Correspondence: lisa_ng@idlabs.a-star.edu.sg; lum_fok_moon@idlabs.a-star.edu.sg

This PDF file includes:

Appendix Figure S1: Page 2

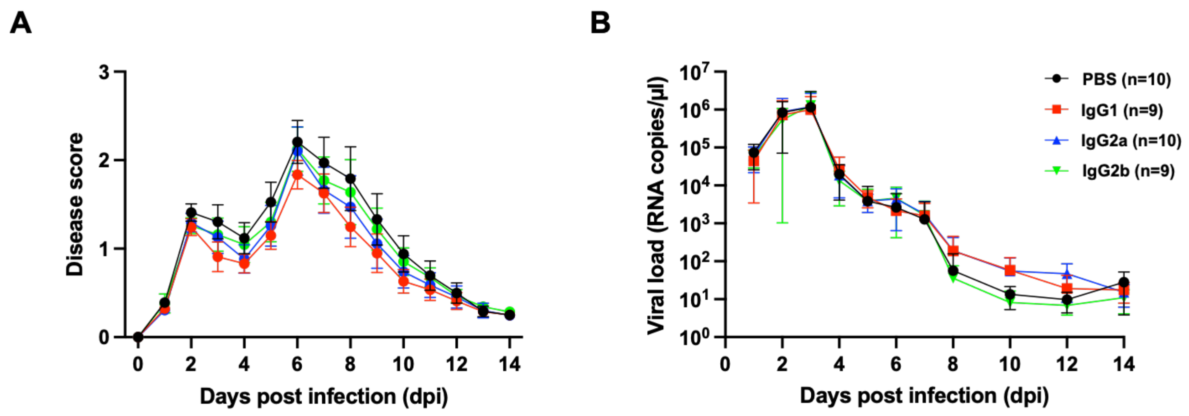

Appendix Figure S1: Treatment of CHIKV-infected animals with IgG1, IgG2b and IgG2a.

(A-B) Wildtype animals were infected with 1 × 10^6 PFU of CHIKV in the right footpad and subsequently treated with rat IgG1 antibodies (0-, 2- and 4-days post-infection (dpi)), rat IgG2b (-1 and 4 dpi) and rat IgG2a (4 dpi). Joint-footpad swelling **(A)** and viral RNA load **(B)** were monitored over 14 days. All data were from biological replicates obtained from two independent experiments. Data are presented as mean ± SD. Data comparisons between the groups receiving antibodies against the PBS control group were performed with non-parametric Mann-Whitney *U* test (two-tailed). No significance was observed.
