# Supplementary material for: Crosstalk between CD64+MHCII+ macrophages and CD4+ T cells drives joint pathology during chikungunya
Source: EMBO Mol Med. 2024 Feb 8;16(3):11. doi: 10.1038/s44321-024-00028-y (PMC10940729; doi:10.1038/s44321-024-00028-y)
Supplement: Supplementary file 8 — Source Data Fig. 6 [file 44321_2024_28_MOESM8_ESM.zip › Figure6/Fig6A/Fig6A README.docx]

Fig6A: ELISPOT images from CD64+MHCII+ replicates and CD64+MHCII- replicates
