# Supplementary material for: Crosstalk between CD64+MHCII+ macrophages and CD4+ T cells drives joint pathology during chikungunya
Source: EMBO Mol Med. 2024 Feb 8;16(3):11. doi: 10.1038/s44321-024-00028-y (PMC10940729; doi:10.1038/s44321-024-00028-y)
Supplement: Supplementary file 12 — Expanded View Figures [file 44321_2024_28_MOESM12_ESM.pdf]

Expanded View Figures

Figure EV1. High-dimensional analysis of mass cytometry data and Immunophenotyping gating strategy.

(**A**) Cluster ID annotation with heatmap. Cluster IDs are indicated in rows, while surface marker expression levels are indicated by the columns. The color represents the relative mean signal intensity of a surface marker. Blue and red represent low and high intensity, respectively. (**B**) Cellular infiltrates in the CHIKV-infected joint-footpad at 6 days post-infection (dpi) were determined with flow cytometry. Briefly, live $CD45^{+}$ immune cells were gated out before the $CD3^{+}$ (inclusive of $CD4^{+}$ and $CD8^{+}$) T cells, $CD3^{+}NK1.1^{+}$ NKT cells, $CD11b^{+}Ly6G^{+}$ neutrophils, $NK1.1^{+}$ NK cells, $CD11b^{+}Ly6C^{+}$ monocytes and $CD64^{+}$ macrophages were identified. Plots shown are representative of a single CHIKV-infected mouse.

© The Author(s)

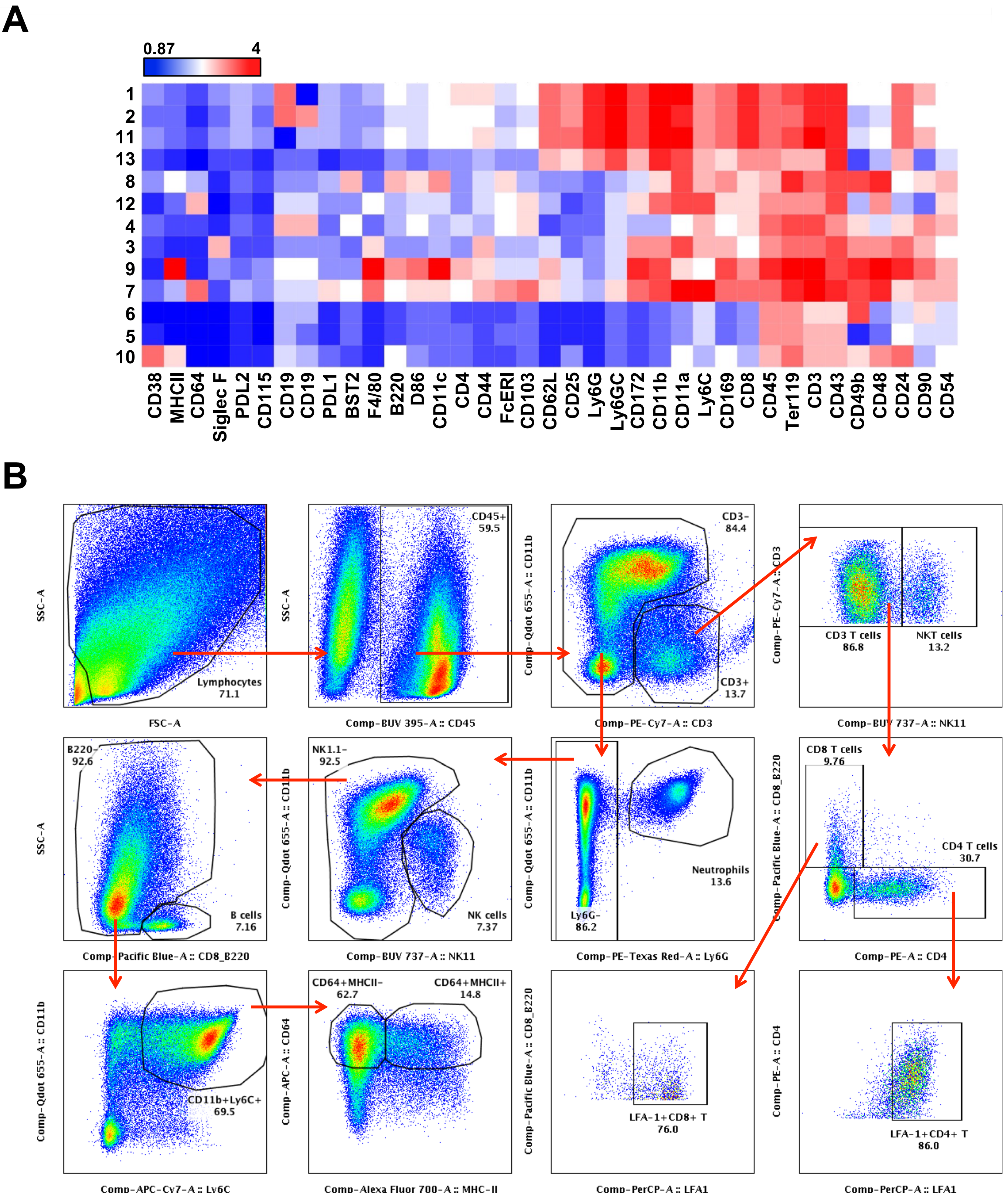
A
0.87
4
1
2
11
13
8
12
4
3
9
7
6
5
10
CD38 MHCII CD64 Siglec F PDL2 CD115 CD19 CD19 PDL1 BST2 F4/80 B220 D86 CD11c CD4 CD44 FcERI CD103 CD62L CD25 Ly6G Ly6GC CD172 CD11b CD11a Ly6C CD169 CD8 CD45 Ter119 CD3 CD43 CD49b CD48 CD24 CD90 CD54
B
SSC-A
FSC-A
Lymphocytes 71.1
Comp-BUV 395-A :: CD45
CD45+ 59.5
Comp-Qdot 655-A :: CD11b
Comp-PE-Cy7-A :: CD3
CD3- 84.4
CD3+ 13.7
Comp-PE-Cy7-A :: CD3
Comp-BUV 737-A :: NK11
CD3 T cells 86.8
NKT cells 13.2
B220- 92.6
B cells 7.16
Comp-Pacific Blue-A :: CD8_B220
NK1.1- 92.5
NK cells 7.37
Comp-BUV 737-A :: NK11
Neutrophils 13.6
Ly6G- 86.2
Comp-PE-Texas Red-A :: Ly6G
Comp-Pacific Blue-A :: CD8_B220
CD8 T cells 9.76
CD4 T cells 30.7
Comp-PE-A :: CD4
Comp-Qdot 655-A :: CD11b
CD11b+Ly6C+ 69.5
Comp-APC-Cy7-A :: Ly6C
Comp-APC-A :: CD64
CD64+MHCII- 62.7
CD64+MHCII+ 14.8
Comp-Alexa Fluor 700-A :: MHC-II
LFA-1+CD8+ T 76.0
Comp-PerCP-A :: LFA1
Comp-PE-A :: CD4
LFA-1+CD4+ T 86.0
Comp-PerCP-A :: LFA1

© The Author(s)

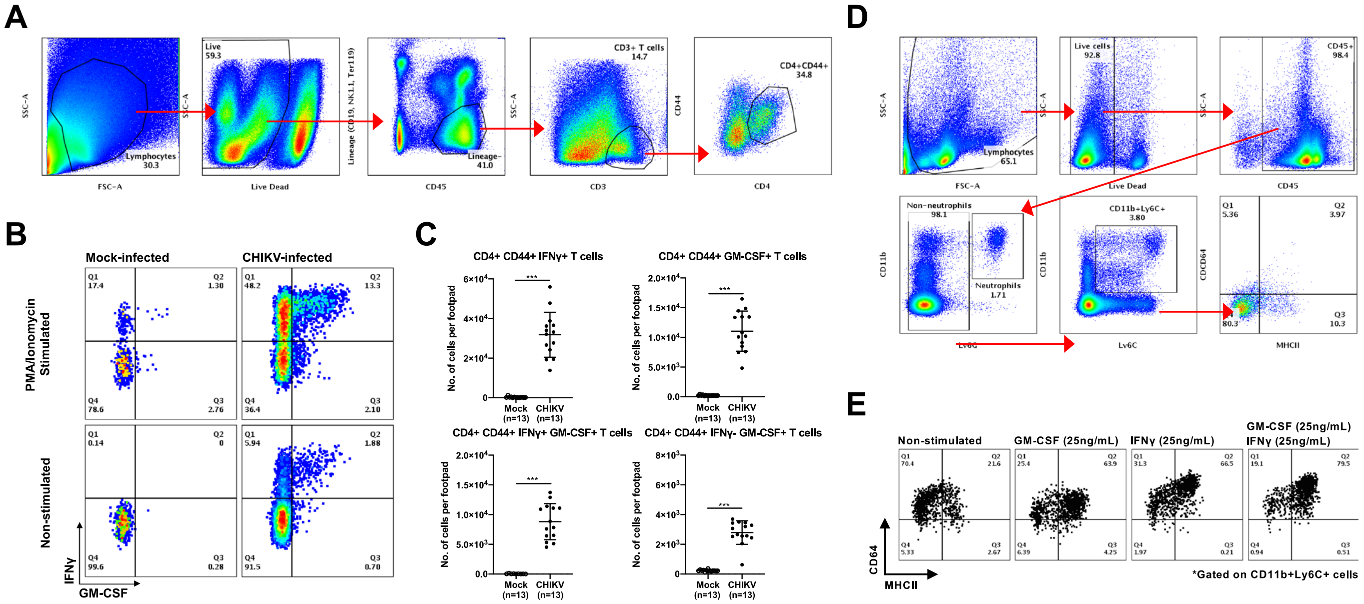

Figure EV2. Intracellular staining reveals presence of IFNγ- and GM-CSF-producing $CD4^+CD44^+$ T cells during CHIKV infection.

(**A**) Joint-footpad cells from non-infected and CHIKV-infected animals were harvested at 6 dpi and were subjected to stimulation with ionomycin and Phorbol Myristate Acetate (PMA) for 4 h. Stimulated cells were subsequently stained for the presence of GM-CSF and IFNγ in $CD4^+CD44^+$ memory/effector T cells. Representative electronic gating strategy to isolate $CD4^+CD44^+$ T cells from joint-footpad cells. Plots shown are concatenated from CHIKV-infected samples. (**B**) Representative flow cytometry plot illustrating the gating strategy in the identification of GM-CSF and IFNγ within the $CD4^+CD44^+$ T cells. (**C**) Dotplots showing the numbers of the various identified subsets within the joint-footpads. Data were from biological replicates obtained from two independent experiments. All data are presented as mean ± SD. Data comparisons between the groups were performed with non-parametric Mann–Whitney *U* test (two-tailed). $IFN\gamma^+$ T cells, $***P = 0.0000000499$; $GM\text{-}CSF^+$ T cells, $***P = 0.0000000499$; $IFN\gamma^+GM\text{-}CSF^+$ T cells, $***P = 0.0000000499$; $IFN\gamma^-GM\text{-}CSF^+$ T cells, $***P = 0.0000000499$. (**D**) Fresh blood was obtained from non-infected animals. Red blood cells were subsequently lysed and live cells were stained with a commercially available live/dead dye before being stained with a cocktail of antibodies targeting surface markers CD45, CD11b, Ly6G, Ly6C, CD64, and MHCII. Live $CD45^+$ cells were firstly identified, and non-neutrophils were gated next. $CD11b^+Ly6C^+$ monocytes were identified from the non-neutrophils and were shown to be low in CD64 and MHCII expression (Q4). Plots shown are representative of a single non-infected mouse. (**E**) Peripheral whole blood was obtained from non-infected animals and were subjected to GM-CSF and IFNγ stimulation after removal of the red blood cells. Stimulated cells were harvested 24 h later and stained for the identification of monocytes and macrophage subsets. Representative flow cytometry plots showing the gating strategy used to identify $CD64^+MHCII^+$ cells among the precursor $CD11b^+Ly6C^+$ monocytes.

 © The Author(s)

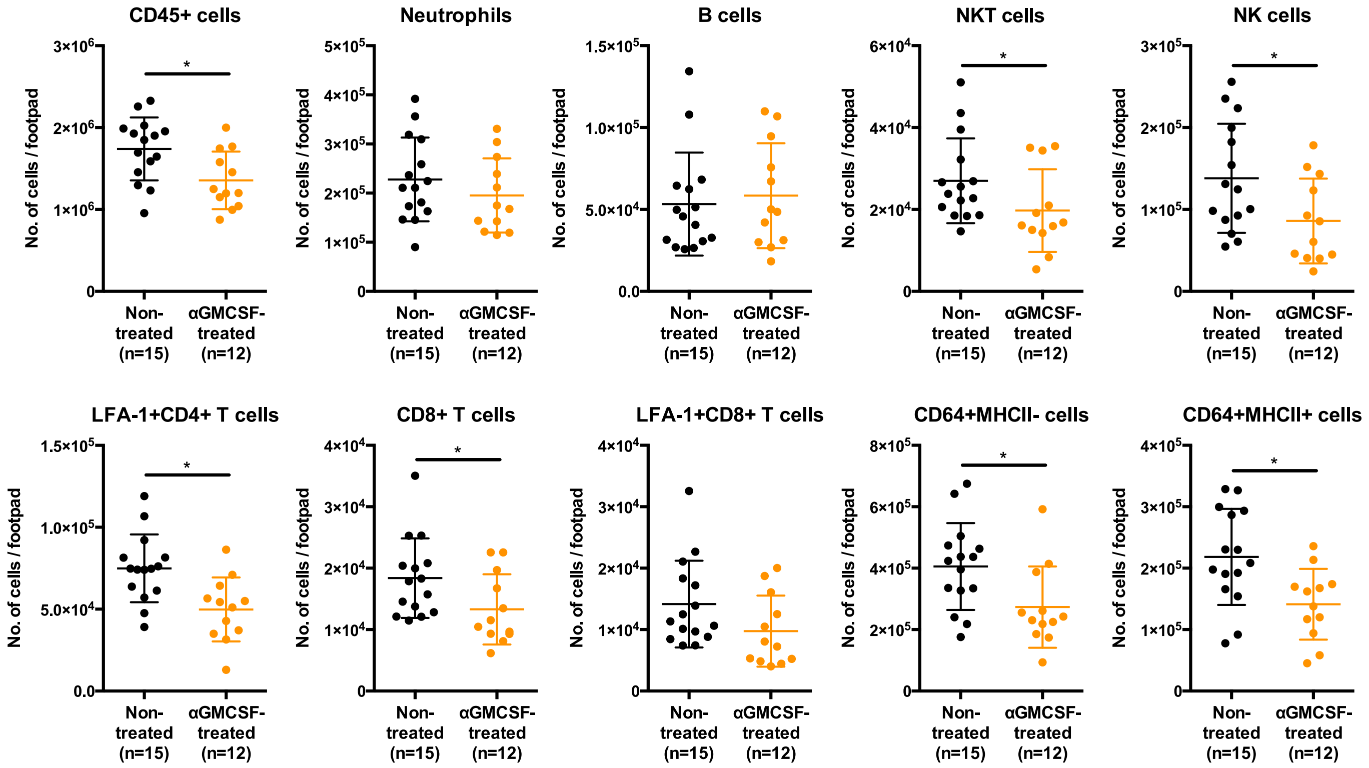

Figure EV3. Depletion of GM-CSF alters the numbers of immune cells infiltrating the joint-footpad.

CHIKV infection (1×10^6 PFU) was performed in the right footpad of wild-type animals. Four days post-infection (dpi), anti-GM-CSF antibodies were given intraperitoneally. Subsequently, numbers of infiltrating $CD45^+$ cells, neutrophils, B cells, NKT cells. NK cells, LFA-1^+CD4^+ T cells, $CD8^+$ T cells, LFA-1^+CD4^+ T cells, $CD11b^+Ly6C^+$ cells, $CD64^+MHCII^-$ cells and $CD64^+MHCII^+$ cells were obtained following immunophenotyping of the joint-footpad at 6 days post-infection (dpi). Data were from biological replicates obtained from two independent experiments. All data are presented as mean ± SD. Data comparisons between the groups were performed with non-parametric Mann–Whitney *U* test (two-tailed): CD45 +, $^*P = 0.0139$; NKT, $^*P = 0.0469$; NK, $^*P = 0.0214$; LFA-1^+CD4^+ T, $^*P = 0.031$; $CD8^+$ T, $^*P = 0.0281$; $CD64^+MHCII^-$, $^*P = 0.0186$; $CD64^+MHCI^+$, $^*P = 0.0161$.

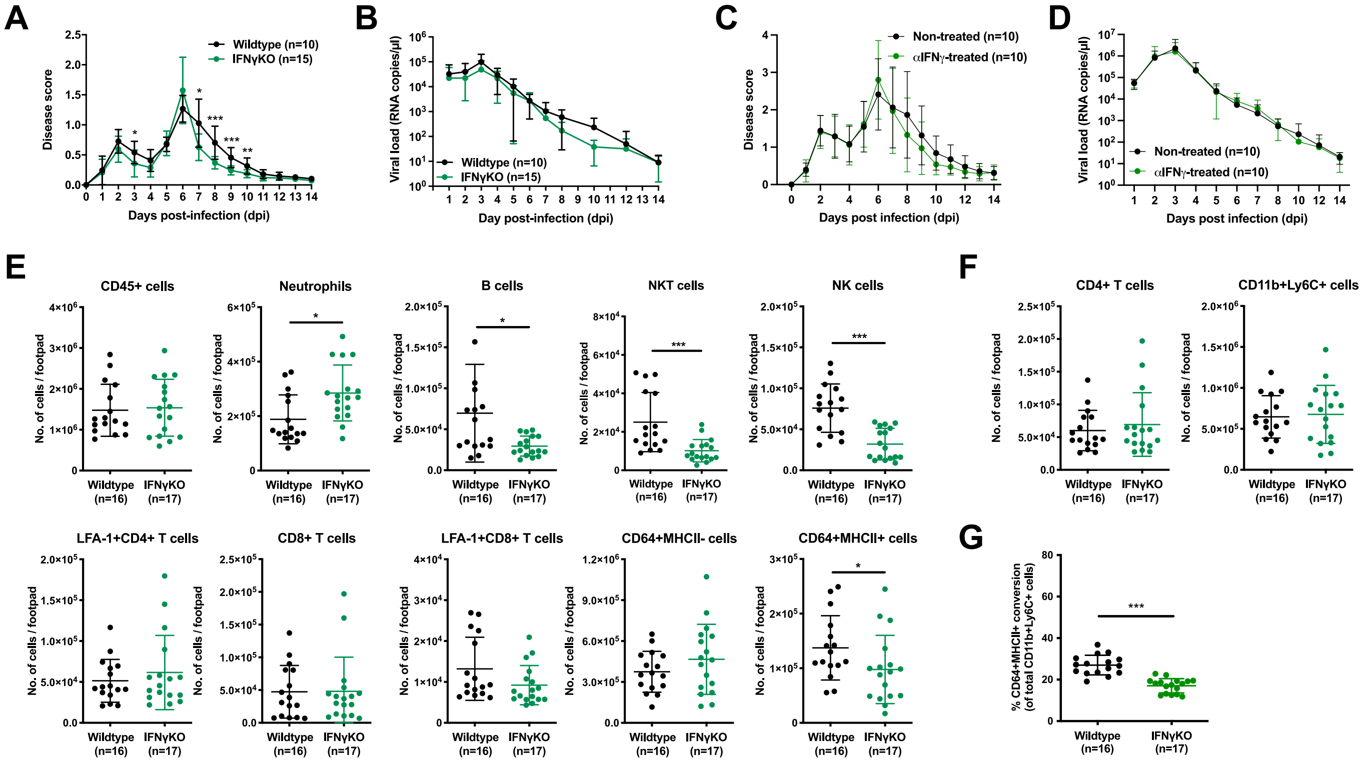

Figure EV4. Absence of IFNγ alters the conversion of CD64⁺MHCII⁺ macrophages in the joint-footpad.

(**A**,**B**) IFNγKO animals were infected with 1 × 10⁶ PFU of CHIKV in the right footpad. Joint-footpad swelling (**A**) and viral RNA load (**B**) were monitored over 14 days. Data were from biological replicates obtained from two independent experiments and presented as mean ± SD. Comparisons between the groups were performed with non-parametric Mann–Whitney *U* test (two-tailed). For footpad swelling: 3 dpi, $*P = 0.0395$; 7 dpi, $*P = 0.0216$; 8 dpi, $***P = 0.00000214$; 9 dpi, $***P = 0.0003$; 10 dpi, $**P = 0.0051$. (**C**,**D**) Wild-type animals were infected with 1 × 10⁶ PFU of CHIKV in the right footpad and were treated with anti-IFNγ antibodies at 0-, 2- and 4-days post-infection (dpi). Joint-footpad swelling (**C**) and viral RNA load (**D**) were monitored over 14 days. Data were from biological replicates obtained from two independent experiments and presented as mean ± SD. (**E**–**G**) Immunophenotyping of IFNγKO joint-footpad was performed at 6 dpi to determine the numbers of infiltrating CD45⁺ cells, neutrophils, B cells, NKT cells. NK cells, LFA-1⁺CD4⁺ T cells, CD8⁺ T cells, LFA-1⁺CD8⁺ T cells, CD64⁺MHCII⁻ cells and CD64⁺MHCII⁺ cells (**E**). Numbers of joint-footpad infiltrating CD4⁺ T cells and CD11b⁺Ly6C⁺ monocytes are depicted (**F**). Percentage differentiation of CD11b⁺Ly6C⁺ monocytes into CD64⁺MHCII⁺ macrophages in CHIKV-infected wild type or IFNγKO animals is shown (**G**). Data were from biological replicates obtained from two independent experiments and presented as mean ± SD. Comparisons between the groups were performed with non-parametric Mann–Whitney *U* test (two-tailed). For neutrophils, $*P = 0.0136$; B cells, $*P = 0.0136$; NKT, $***P = 0.0002$; NK, $***P = 0.0001$; CD64⁺MHCII⁺, $*P = 0.0207$; for % CD64⁺MHCII⁺ conversion (**G**), $***P = 0.000000105$.

 © The Author(s)

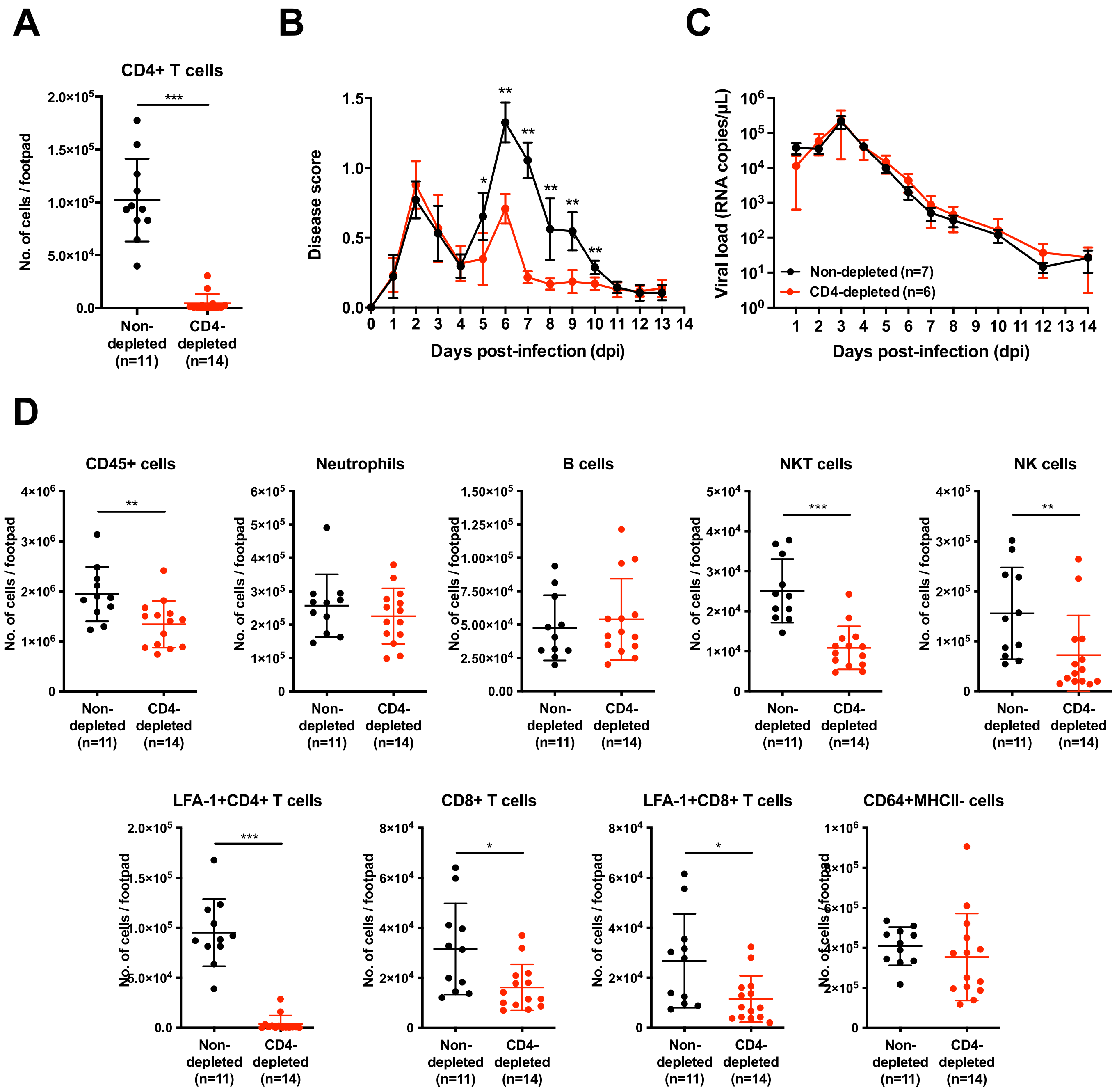

Figure EV5. CD4-depletion reduces CHIKV disease severity.

(**A**) Wild-type animals were infected with 1 × 10^6 PFU of CHIKV in the right footpad and anti-CD4 antibodies were given intraperitoneally at -1 and 4 days post-infection (dpi). Immunophenotyping of joint-footpad was performed at 6 dpi to determine the numbers of infiltrating $CD4^+$ T cells. Data were from biological replicates obtained from two independent experiments and presented as mean ± SD. Data comparisons between the groups were performed with non-parametric Mann–Whitney *U* test (two-tailed). $CD4^+$ T cells, ***$P = 0.000000449$. (**B**,**C**) Joint-footpad swelling (**B**) and viral RNA load (**C**) of CHIKV-infected animals with or without administration of anti-CD4 antibodies, over a period of 14 days. Data were from biological replicates obtained from two independent experiments and presented as mean ± SD. Data comparisons between the groups were performed with non-parametric Mann–Whitney *U* test (two-tailed). For footpad swelling: 5 dpi, *$P = 0.0192$; 6 dpi, **$P = 0.0012$; 7 dpi, **$P = 0.0012$; 8 dpi, **$P = 0.0012$; 9 dpi, **$P = 0.0012$; 10 dpi, **$P = 0.0023$. (**D**) Numbers of infiltrating $CD45^+$ cells, neutrophils, B cells, NKT cells. NK cells, LFA-1^+CD4^+ T cells, $CD8^+$ T cells, LFA-1^+CD4^+ T cells, $CD11b^+Ly6C^+$ cells and $CD64^+MHCII^-$ cells were determined following immunophenotyping of the joint-footpad at 6 dpi (**A**). Data were from biological replicates obtained from two independent experiments and presented as mean ± SD. Data comparisons between the groups were performed with non-parametric Mann–Whitney *U* test (two-tailed). $CD45^+$, **$P = 0.0042$; NKT, ***$P = 0.0000306$; NK, **$P = 0.0075$; LFA-1^+CD4^+ T, ***$P = 0.000000449$; $CD8^+$, *$P = 0.0108$; LFA-1^+CD8^+ T, *$P = 0.0179$.
